# A cross-sectional online survey of quality of life in adults with Klinefelter syndrome in the UK

**DOI:** 10.1530/EC-26-0419

**Published:** 2026-10-12

**Authors:** Brien Mehmet, Andrew A Dwyer, Channa N Jayasena, Steve Gillard, Sofia Llahana

**Affiliations:** ^1^ School of Health & Medical Sciences, City St George’s, University of London, London, United Kingdom; ^2^ Department of Endocrinology and Diabetes, Royal Free Hospital NHS Foundation Trust, London, United Kingdom; ^3^ Boston College William F. Connell School of Nursing, Chestnut Hill, Massachusetts, USA; ^4^ P50 Massachusetts General Hospital – Harvard Center for Reproductive Medicine, Boston, Massachusetts, USA; ^5^ Department of Investigative Medicine, Imperial College London, Hammersmith, London, United Kingdom

**Keywords:** quality of life, Klinefelter syndrome, reproductive endocrinology, patient-reported outcomes

## Abstract

Klinefelter syndrome (KS) is the most common sex chromosome disorder in males and the leading genetic cause of hypogonadism, affecting approximately 1 in 450–600 male births. Despite its prevalence, many individuals remain undiagnosed. Although European cohort studies have demonstrated poorer health-related quality of life (HR-QoL) among individuals with KS, evidence from the United Kingdom remains limited. This cross-sectional online survey explored HR-QoL and perceived self-efficacy in clinical communication among adults with KS in the United Kingdom. Sixty adults with a confirmed diagnosis of KS were recruited through the Klinefelter Syndrome Association (UK). Participants completed validated measures of HR-QoL (WHOQOL-BREF) and Perceived Efficacy in Patient–Physician Interactions (PEPPI-5), alongside clinical and sociodemographic questions. Participants reported a broad range of physical and psychological comorbidities, with 32 (53%) of participants self-reporting depression and/or anxiety. Compared with the UK normative data (*n* = 141), participants with KS demonstrated significantly lower HR-QoL scores across all WHOQOL-BREF domains. Linear regression analyses showed that greater perceived self-efficacy in clinician–patient communication was associated with better HR-QoL outcomes across all WHOQOL-BREF domains after adjustment for body mass index, comorbidity count, and self-reported depression and/or anxiety, with adjusted coefficients ranging from *B* = 4.06 to 6.98 WHOQOL points per 5-point increase in PEPPI-5 (*P* ≤ 0.022 for all domains). These findings highlight the substantial impact of KS on quality of life and emphasise the importance of interventions aimed at improving communication, supportive care, and patient engagement to enhance HR-QoL outcomes in adults with KS.

## Introduction

Klinefelter syndrome (KS) is the most common sex chromosome aneuploidy identified in males (1). KS, characterised by the 47,XXY karyotype, although estimates vary, affects approximately 1 in 450–600 male births. Despite its prevalence, KS remains substantially underdiagnosed, and evidence suggests 50–75% of affected people never receive a diagnosis (2, 3, 4, 5). Notably, individuals with KS are diagnosed in adulthood, resulting in delayed access to endocrine, fertility, and psychosocial services. The exact number of individuals diagnosed with KS in the UK is not readily available. However, based on the published prevalence estimates, approximately 57,500 to 76,700 people may be living with KS in the United Kingdom (UK), with an estimated 50–75% of affected people never receiving a correct diagnosis (3). However, UK-specific data and evidence on health-related quality of life (HR-QoL) outcomes remain limited.

Common clinical features in KS include hypergonadotropic hypogonadism, infertility, gynaecomastia, tall stature, eunuchoid body proportions, and cardiometabolic comorbidities. There is also a range of associated cognitive, neurodevelopmental, and psychological features, including learning difficulties, executive functioning difficulties, social-cognitive challenges, and a higher prevalence of depression and anxiety (3, 6, 7, 8, 9). The constellation of symptoms experienced significantly affects physical, psychological, and social well-being outcomes and the overall HR-QoL in individuals with KS (10). The increased burden of comorbidity associated with KS contributes to a 70% greater risk of hospitalisation and a 50% increased mortality rate (11). The varied signs, symptoms, and outcomes highlight KS as a complex condition with lifelong impact that extends beyond endocrine and reproductive health.

Management of KS requires individualised, multidisciplinary care. Testosterone replacement therapy (TRT) is commonly used to treat hypogonadism and is recommended in current clinical guidance (12) due to beneficial effects on body composition and bone mineral density (13). However, research on the benefits of TRT beyond body composition and bone health, including HR-QoL, is less consistent and may depend on treatment modality, dosage, and adherence, as well as underlying comorbidities. Testicular dysfunction and infertility are common features of KS and can have a considerable emotional and psychological impact on men living with the condition. While most adults with KS have azoospermia or severe impairment of spermatogenesis, assisted reproductive techniques, including conventional or microdissection testicular sperm extraction followed by intracytoplasmic sperm injection, may provide an opportunity for biological parenthood in some individuals with non-obstructive azoospermia, including individuals with KS (14). Discussions surrounding fertility preservation and assisted reproduction are often complex and emotionally challenging. Uncertainty regarding fertility potential, together with the variable success of assisted reproductive treatments, contributes to anxiety, distress, and concerns about future parenthood. Given the wide-ranging physical, emotional, and psychosocial impacts of KS, HR-QoL patient-centred outcomes are key indicators of the effectiveness of healthcare support and services.

Large multinational cohort studies involving people with KS reveal significant HR-QoL deficits, highlighting the burden of comorbidities, neurodevelopmental differences, and the lived experiences of individuals with KS (15, 16). However, UK-based data on factors associated with HR-QoL remain limited. This is important because healthcare and specialist access, service infrastructure, and other support systems may vary between countries and may influence HR-QoL outcomes. Therefore, generating evidence from different national contexts, including the UK, will contribute to a broader international evidence base, helping to identify HR-QoL deficits that are consistent across countries and those that may be country-specific. In addition, validated HR-QoL scales designed for general populations may overlook KS-specific phenomena and may not reflect the lived experiences of individuals with KS (17).

Healthcare experiences may represent an important factor associated with HR-QoL in individuals with KS. In a large qualitative research study of 310 participants, the number of respondents to each open-ended question ranged from 169 to 210. Over half of respondents reported difficulty in finding providers knowledgeable about KS. It was also reported that participants felt unsupported and uninformed, highlighting broader health-system challenges and possibly fragmented care pathways (18). These challenges may hinder continuity of care and engagement with long-term management plans. Research on other chronic conditions has linked patient confidence in navigating clinical encounters, their ability to express concerns, ask questions important to them, and have clinicians take them seriously to improved outcomes, with shared decision-making, adherence, and access to appropriate care being mechanisms to improved outcomes (19, 20). However, these mechanisms have received limited attention in KS populations, especially in a UK setting. To address these gaps, we aimed to explore HR-QoL outcomes in individuals with KS in the UK and examine patient–provider interactions as a potential mediator of HR-QoL.

## Methods

We used a cross-sectional online survey to investigate HR-QoL in KS. The City St George's, University of London ethics committee approved the study (ETH2223-1914). Participants were asked to complete an eligibility checklist prior to proceeding with the survey; as data were anonymous, survey completion indicated informed consent. Study findings are reported using STROBE guidelines for cross-sectional studies (21).

Adults (18 years or older) residing in the UK with a confirmed clinical diagnosis of KS and who were able to read/write English were included in the study. Although participants were encouraged to seek assistance from a family member or friend to complete the survey, they were asked that their responses reflect the participants’ own views and experiences.

Participants were recruited between May 2024 and July 2025 through the Klinefelter’s Syndrome Association (KSA) using their membership mailing list, local patient meetings and support groups, and social media posts in KS-relevant networks. As recruitment materials were distributed by collaborating organisations, rather than the research team, the number of individuals who received or viewed the study invitation was not available. Therefore, a formal participation rate could not be calculated. Participants were informed that recruitment was facilitated by the KSA, as collaborators in this study, and that the research team donated £2 to the KSA for every response, as remuneration directly to participants was not possible, as the survey was anonymous.

### Survey instruments

Participants provided self-reported information on their age, ethnicity, education, marital and employment status, comorbidities, TRT use, and fertility history, and completed several validated questionnaires. The Perceived Efficacy in Patient–Physician Interaction (PEPPI-5) scale (22) was used to measure participants’ self-perceived efficacy in healthcare provider communications. The validated instrument includes 5 Likert-type questions that are scored on a 5-point scale, with higher scores indicating greater perceived efficacy. The PEPPI-5 was selected as it is a pre-existing validated instrument with established psychometric properties and has been used in several research studies. The 26-item WHOQOL-BREF (23) was used to measure HR-QoL outcomes along four domains: physical health (7 items, e.g. pain, energy, sleep, mobility, daily activities, and work capacity), psychological health (6 items, e.g. positive/negative feelings, self-esteem, body image, and thinking/learning), social relations (3 items: personal relationships, social support, and sexual activity), and environment (8 items: safety, home environment, finances, access to health care, information, leisure, physical environment, and transport). The WHOQOL-BREF was selected as there are currently no validated KS-specific HR-QoL instruments. Furthermore, it has established psychometric properties in a UK population, allowing comparisons with UK normative population data, and provides an extensive multidimensional assessment of HR-QoL. Two further items assess overall QoL and general health. Items are scored using a 5-point Likert scale, and negatively phrased items are reverse-scored. Higher scores indicate greater HR-QoL in a given domain (range: physical = 7–35, psychological = 6–30, social = 3–15, environment = 8–40). Domain scores were calculated according to the WHO guidelines by averaging item scores within each domain and transforming them to the standard 0–100 scale aiding interpretability and allowing for comparisons with normative data. In addition, internal consistency was assessed using Cronbach’s alpha for each domain.

### Statistical analysis

Data quality screening was based on the following: i) implausibly short response times; ii) patterned responding, such as highly regular or invariant response patterns across Likert items; and iii) invalid open-ended responses, including irrelevant or nonsensical responses, and clearly non-study-related text indicative of automated responses. We excluded 8 cases that met these criteria, resulting in a final sample of 60 participants. Following data cleaning, analyses were completed using IBM SPSS Statistics (Version 29). Data are reported using descriptive statistics and WHOQOL-BREF domain means (SD), and reliability estimates are reported. Forest plots were produced in Python using matplotlib v3.10.7.

WHOQOL-BREF scores from individuals with KS were compared with previously published UK reference data from a ‘*well*’ comparison group (*n* = 141) reported by Skevington and McCrate (24). These participants were not recruited as controls for the present study; instead, their published WHOQOL-BREF domain scores were used for comparative analysis against the current study cohort. Comparisons were completed using two-sample Welch *t*-tests derived from reported group means, standard deviations, and sample sizes. We reported group means (SD), mean differences with 95% confidence intervals (CIs), *P*-values, and standardised mean differences (Cohen’s *d* with 95% CIs). *P*-values are presented without multiplicity adjustment.

We used linear regression modelling to evaluate the relationship between perceived self-efficacy in patient–physician interactions, measured using the PEPPI-5 and HR-QoL (*n* = 60). Covariates were selected *a priori* based on the clinical relevance to KS and previous evidence suggesting that BMI, mental health status, and comorbidity burden are associated with adverse HR-QoL outcomes (25, 26, 27). Two models were created: i) unadjusted regression analysis with PEPPI-5 total score as the sole predictor (*n* = 60) and WHOQOL-BREF domain scores (physical, psychological, social relationships, and environment) as dependent variables; and ii) an adjusted regression analysis including PEPPI-5 total score, BMI (kg/m^2^), self-reported depression and anxiety (yes/no), and continuous comorbidity count (*n* = 55) as covariates, with WHOQOL-BREF domains as dependent variables. PEPPI-5 total score was scaled per 5-point increase. Comorbidity count was calculated for each participant by summing the number of self-reported comorbidities. Each reported condition was counted once, and the resulting total was treated as a continuous variable. Self-reported depression and anxiety were included as clinically relevant covariates to account for the impact of mental-health status in the adjusted models. However, for the psychological health domain, depression and anxiety were considered closely related to the outcome construct instead of independent confounders. In this case (psychological health), adjusted estimates should be interpreted as conditional associations.

Model assumptions were assessed using variance inflation factors (VIFs) and condition indices, Durbin–Watson statistic tests, and standardised residual diagnostics, including histograms, normal P–P plots and standardised residuals versus standardised fitted values. Diagnostic statistics and residual plots indicated no major violation of model assumptions.

### Content analysis of free text responses

Open-ended responses were analysed using inductive content analysis (28) to generate and quantify themes describing participants’ experiences, which were illustrated with example quotations.

## Results

Participant demographics are presented in Table 1. Most participants (68.3%) were between 25 and 54 years old, and the majority identified as being White (79.6%); mean age at diagnosis was 25.1 ± 16.1 years. Overall, participants had attained a good educational level, with 36.7% having completed an undergraduate degree, 21.7% a postgraduate degree, 75% were employed full-time, and 48.3% were married. The age distribution of participants is presented in Fig. 1.

**Table 1:** Sociodemographics of study participants with Klinefelter syndrome (*n* = 60). Values are presented as *n* (%) or as mean ± SD. Gender identity categories describe the characteristics of the current cohort and should not be interpreted as prevalence estimates for the wider KS population.

Characteristics	Values
Gender identity	
Male	51 (85.0%)
Female	5 (8.3%)
Non-binary/third gender	2 (3.3%)
Prefer not to say	1 (1.7%)
Intersex	1 (1.7%)
Self-reported race (*n* = 59)	
White	47 (79.7%)
Black/African/Caribbean	8 (13.6%)
Asian	2 (3.4%)
More than one race	2 (3.4%)
Education level	
Did not obtain GCSE or O level	5 (8.3%)
Secondary school/high school	20 (33.3%)
Undergraduate degree	22 (36.7%)
Postgraduate degree	13 (21.7%)
Marital status	
Single	16 (26.7%)
Married	29 (48.3%)
In a relationship	9 (15%)
Divorced	3 (5%)
Widowed	1 (1.7%)
Prefer not to say	2 (3.3%)
Employment status	
Employed	45 (75%)
Long-term unemployment	2 (3.3%)
Student	5 (8.3%)
Retired	7 (11.7%)
Prefer not to say	1 (1.7%)
Physical status	
BMI, kg/m^2^	28.8 ± 5.9

**Figure 1:**
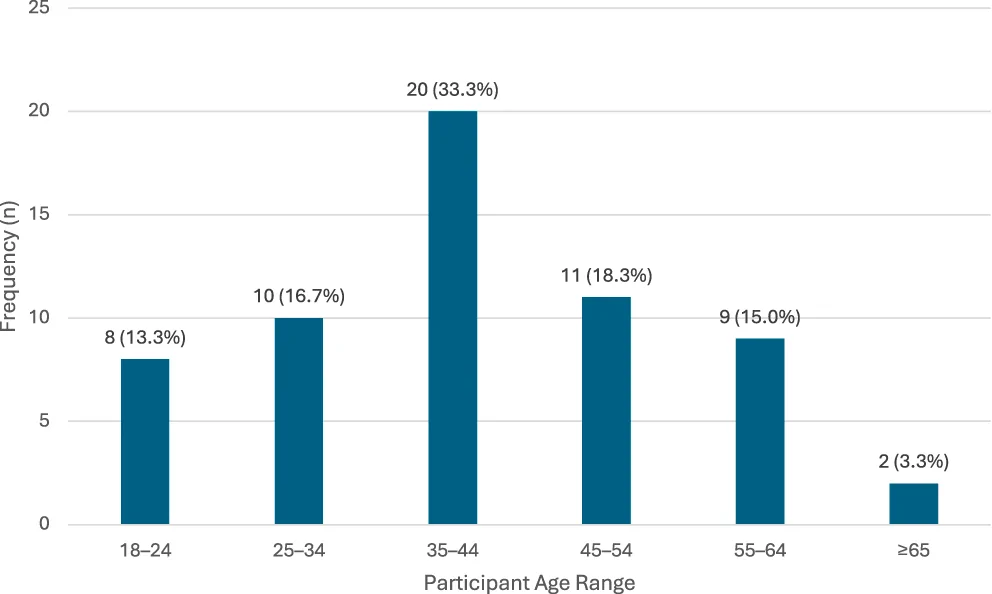
Age distribution of adults with Klinefelter syndrome (*n* = 60). Bar chart showing numbers and percentages of participants in each age group. The largest group was aged 35–44 years, representing 20 of 60 participants (33.3%).

Information relating to testosterone replacement therapy (TRT) use and parenthood is presented in Table 2. Nearly three-quarters (73.3%) reported current TRT use, with 52.3% of TRT users receiving long-acting injectable formulations. Most participants (78.3%) did not have children. Among participants who reported having children, one reported conception through assisted fertility treatment. Twenty-two participants reported having attempted fertility treatments. Our survey did not collect detailed information regarding the type of fertility treatment such as TESE or micro-TESE, the number of unsuccessful attempts, and treatment outcomes.

**Table 2:** Testosterone replacement therapy (TRT) and fertility demographics of the Klinefelter syndrome participants (*n* = 60). Values are presented as *n* (%).

Variable	Values
TRT	
Yes	44 (73.3%)
No	16 (26.7%)
If no, were you offered TRT?	
Yes	9
No	7
Testosterone formulations used	
Long-acting injection	23
Topical	16
Short-acting injection	5
Do you have children?	
Yes	12 (20%)
No	47 (78.3%)
Prefer not to say	1 (1.7%)
If yes (*n* = 12)	
Conceived naturally	1
Adopted	3
Assisted fertility	1
Sperm donation	7
Attempted fertility treatment	
Yes	22 (36.7)
No	36 (60%)
Prefer not to say	2 (3.3%)

### Comorbidities

As depicted in Fig. 2, self-reported comorbidities are presented with depression and/or anxiety being the most frequently reported (53%), followed by poor sleep quality (38%), gynaecomastia (35%), cardiometabolic disease (35%), and neurodevelopmental disorders (33%). Adverse bone health (23%), respiratory disease (20%), sleep apnoea (15%), thrombosis (13%), pulmonary embolism (10%), and musculoskeletal conditions (8%) were also reported. Less frequent comorbidities included varicose veins (8%), anaemia (5%), cancers of all types (5%), autonomic syndromes (5%), chronic fatigue (3%), cardiovascular disease (3%), bipolar disorder (3%), and several conditions each reported by one participant, including ophthalmological and gastrointestinal disorders, sacral implant, thrombocytopenia, and kidney dysplasia (all 2%).

**Figure 2:**
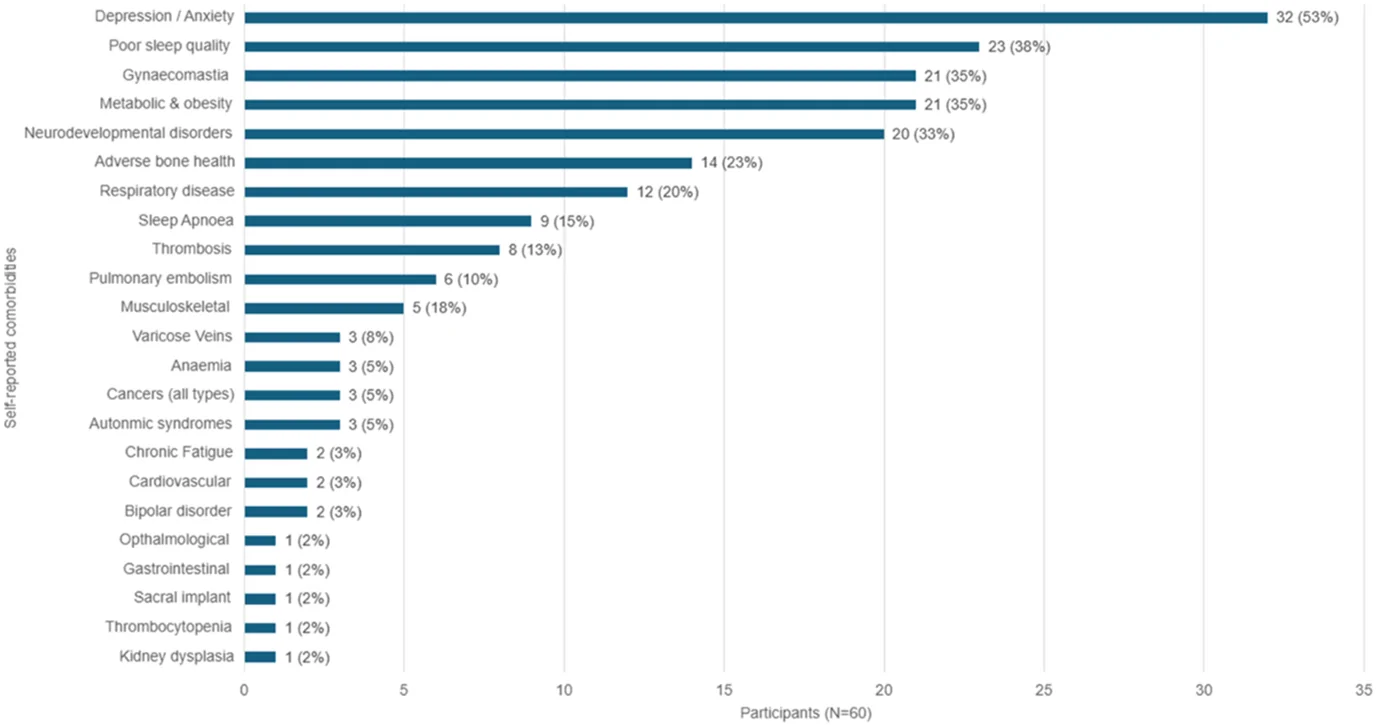
Self-reported comorbidities in adult participants with Klinefelter syndrome (*n* = 60). Bar chart showing the frequency and percentages of self-reported comorbidities among survey participants (*n* = 60). Participants could report more than one condition. Neurodevelopmental disorders included self-reported autism spectrum condition (*n* = 6), dyslexia (*n* = 9), dyspraxia (*n* = 3), Irlen syndrome (*n* = 1), attention deficit hyperactivity disorder (*n* = 5), and an unspecified neurodevelopmental disorder diagnosis (*n* = 5). Categories were not mutually exclusive.

### Health-related quality of life

Internal consistency using Cronbach’s alpha demonstrated acceptable-to-good reliability for physical health (*α* = 0.858), social relations (*α* = 0.746), and environment (*α* = 0.848) domains, but lower internal consistency for psychological health (*α* = 0.576) domains.

#### Participants with KS had worse HR-QoL scores compared with normative data

Participants with KS (*n* = 60) reported significantly lower HR-QoL across all domains compared with the UK normative data (*n* = 141) (all *P* < 0.001). Mean (SD) scores for participants with KS versus UK normative values were 53.50 ± 5.92 vs 75.41 ± 18.72 for physical health, psychological health 51.97 ± 11.50 vs 70.21 ± 15.84, social relationships 53.78 ± 14.56 vs 71.37 ± 19.82, and environment 60.47 ± 17.95 vs 72.26 ± 14.78. Standardised mean differences reveal large negative effect sizes for physical health (*d* = −1.37; 95% CI: −1.70, −1.04), psychological health (*d* = −1.24; 95% CI: −1.57, −0.92), and social relationships (*d* = −0.96; 95% CI: −1.27, −0.64) HR-QoL, with smaller but still a medium-to-large effect for the environmental domain (*d* = −0.75; 95% CI: −1.06, −0.44). All confidence intervals remained below zero (Fig. 3), indicating consistently lower HR-QoL scores among participants with KS.

**Figure 3:**
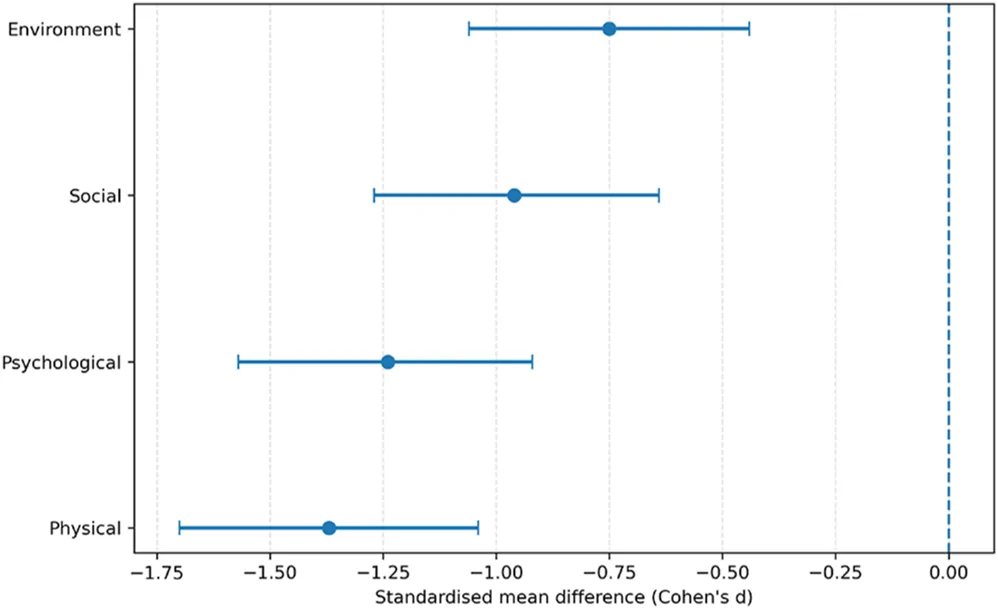
Forest plot highlighting standardised mean differences (*d*) in individuals with KS (*n* = 60) vs the UK normative data (*n* = 141). Forest plot showing standardised mean differences, Cohen’s *d* reported between adults with Klinefelter syndrome (*n* = 60) and pre-published UK ‘healthy adult’ data (*n* = 141) across WHOQOL-BREF domains. The dashed line at zero indicates no differences between groups; negative values show lower HR-QoL among adults with Klinefelter syndrome.

#### Quality of life and testosterone replacement therapy

No significant differences in HR-QoL were observed between participants receiving TRT and those not receiving TRT. Standardised mean differences were small to moderate across all domains. Including physical health (*d* = 0.49, *P* = 0.10), psychological health (*d* = 0.36, *P* = 0.20), social relations (*d* = −0.09, *P* = 0.35), and environment (*d* = 0.35, *P* = 0.23). Figure 4 illustrates wide confidence intervals for all domains, each crossing zero, indicating imprecision and no statistically significant between-group differences. The plot suggests a non-significant nominal difference favouring TRT in the physical domain; however, effects are inconsistent, and all confidence intervals crossed zero.

**Figure 4:**
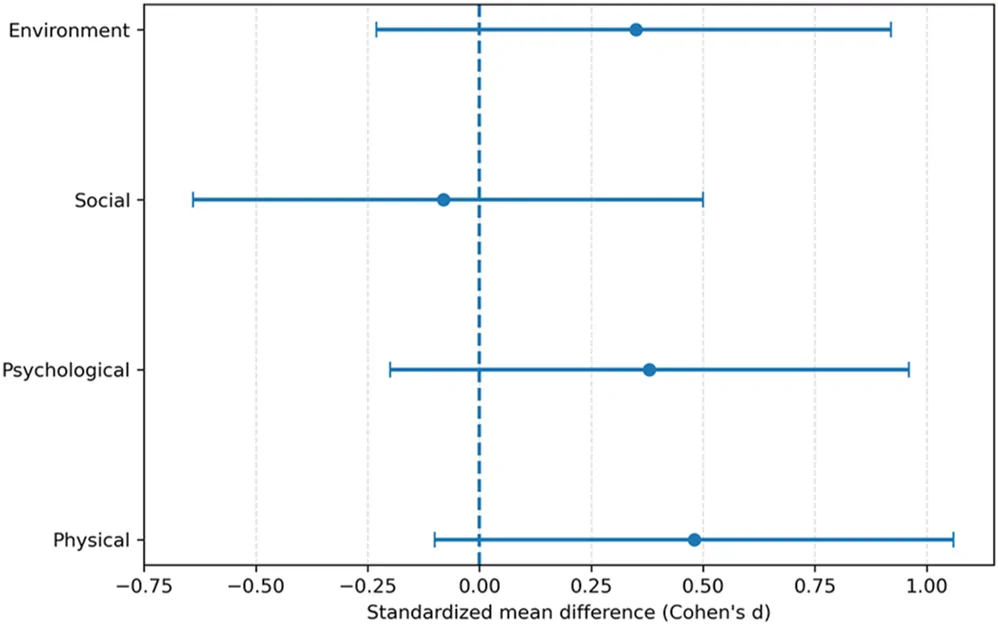
Forest plot highlighting standardised mean differences (*d*) in individuals with KS using TRT (*n* = 44) vs participants currently not using TRT (*n* = 16). Forest plot showing standardised mean differences, Cohen’s *d* reported between adults with Klinefelter syndrome currently using testosterone replacement therapy (TRT; *n* = 44) and those not currently using TRT (*n* = 16). The vertical line at zero indicates that there is no difference between the groups.

#### Communication and self-efficacy with clinicians

The PEPPI-5 was a reliable measure of perceived self-efficacy in patient–physician interactions among adults with KS, with all five items demonstrating good internal consistency (Cronbach’s *α* ≥ 0.86). The mean PEPPI-5 score in this sample was 14.52 ± 5.42 (range: 5–25), suggesting low levels of self-efficacy in clinical interactions (Table 3). Participants reported the greatest confidence in knowing what questions to ask clinicians, whereas the lowest scores related to perceived confidence that a clinician would take their chief health concerns seriously.

**Table 3:** Perceived self-efficacy in patient–physician interactions in patients with KS (*n* = 60). Values are presented as mean ± SD.

Item	Values
‘How confident are you in your ability to…’	
Know what questions to ask a clinician	3.37 ± 1.35
Get a clinician to answer all of your questions	2.87 ± 1.42
Make the most of your visit with the clinician	2.93 ± 1.37
Get a clinician to take your chief health concerns seriously	2.62 ± 1.38
Get a clinician to do something about your chief health concern	2.73 ± 1.33
Total PEPPI-5 scores	14.52 ± 5.42

### Patient–physician interactions and quality of life

Table 4 presents the unadjusted and adjusted linear regression models examining associations between PEPPI-5 total scores and WHOQOL-BREF domain scores.

**Table 4:** Associations between PEPPI-5 and WHOQOL domain scores with/without adjustment for BMI and depression/anxiety.

Domain/model	Adj. *R^2^*	PEPPI-5		BMI		Depression/anxiety		Comorbidity count	
		B (95% CI)	*P*	B (95% CI)	*P*	B (95% CI)	*P*	B (95% CI)	*P*
Physical health									
Unadjusted (*n* = 60)	0.238	5.31 (2.90–7.73)	<0.001	-	-	-	-		
Adjusted (*n* = 55)	0.304	4.06 (1.37–6.77)	0.004	0.06 (−0.42 to 0.55)	0.24	−5.69 (−11.98 to 0.61)	0.076	−0.75 (−2.00 to 0.69)	0.238
Psychological health									
Unadjusted (*n* = 60)	0.154	5.51 (2.29–8.73)	0.001	-	-	-	-		
Adjusted (*n* = 55)	0.273	4.13 (0.83–7.43)	0.015	−0.24 (−0.832 to 0.354)	0.422	−12.48 (−20.15 to −4.81)	0.002	1.47 (−0.06 to 3.00)	0.059
Social relationships									
Unadjusted (*n* = 60)	0.089	7.56 (1.75–13.37)	0.012	-	-	-	-		
Adjusted (*n* = 55)	0.331	6.98 (1.03–12.93)	0.022	0.84 (−0.23 to 1.91)	0.119	−20.70 (−34.54 to −0.88)	0.004	−1.64 (−4.40 to 1.12)	0.119
Environment									
Unadjusted (*n* = 60)	0.222	8.03 (4.23–11.84)	<0.001	-	-	-	-		
Adjusted (*n* = 55)	0.367	6.19 (2.43–9.95)	0.002	−0.094 (−0.77 to 0.58)	0.781	−10.21 (−18.95 to −1.47)	0.023	−0.02 (−1.77 to 1.72)	0.979

B = unstandardised coefficient; effects are in WHOQOL points per 5-point higher PEPPI-5, per 1 kg/m^2^ BMI, self-reported depression/anxiety coded (yes/no), and per one additional comorbidity. Adj. *R*^2^ pertains to the model shown in the corresponding row.

In unadjusted models, higher PEPPI-5 scores are significantly associated with greater HR-QoL in all domains. Following adjustment, associations were attenuated yet remained significant across all domains, indicating that each 5-point increase in PEPPI-5 corresponded with a 4- to 7-point improvement in WHOQOL-BREF scores (*P* < 0.05 for all domains), with the largest reported for social and environmental domains. Self-reported depression and anxiety had a significant negative association with psychological health, social relations, and environmental domains. Comorbidity count and BMI showed no significant association with any domain. However, comorbidity count showed a borderline association with the psychological health domain (*P* = 0.059).

Model diagnostics indicated low collinearity and acceptable residual behaviour. The case with the highest Cook’s D was removed, and the analysis was re-run; ΔB% (absolute ΔB in units) was calculated (Supplementary materials (see section on Supplementary materials given at the end of the article)). Overall, no conclusions changed after removing the most influential case. The model fit (Adj. *R*^2^) was minimally influenced; findings can, therefore, be considered robust.

### Free text responses

Content thematic analysis was carried out from responses by 50 (83.3%) participants to an open-ended question: ‘Feel free to share how Klinefelter’s syndrome/XXY has impacted your quality of life, well-being, or any other aspects, along with any additional comments or experiences not covered in this questionnaire.’ The analysis revealed six key themes (Table 5). The most common theme was related to mental health (*n* = 14), highlighting adverse psychological outcomes participants experienced living with KS (i.e. anxiety, depression, feelings of helplessness). Another common theme related to ‘medical experiences’ described limited clinical awareness of KS, misdiagnosis, inconsistent care, and frustration with the health system. ‘Infertility and family planning’ (*n* = 6) relates to the sense of grief from an infertility diagnosis. Participants exhibited negative emotions, fear, and feelings of inferiority, alongside challenges to assessing fertility interventions (delays and unsuccessful attempts). Infertility also raised identity concerns, shaping self-concepts related to masculinity, gender norms, and social stigma associated with a lack of parenthood. Participants reported that KS was often diagnosed in infertility clinics. Another theme was ‘positive outlook and acceptance’ (*n* = 9), reflecting the positive impact having a diagnosis has had on HR-QoL through acceptance, sense of community, adaptations, and a positive mindset. ‘Cognitive/learning difficulties’ (*n* = 5) describes learning difficulties throughout life, and challenges with memory and concentration, as well as academic struggles. Participants categorised into the theme ‘fatigue/low energy’ described feeling chronically tired with a reduced vitality that limits leisure activities and activities of daily living. Quotations illustrating each of the six themes are provided in Table 5.

**Table 5:** Content thematic analysis of open-ended questions.

Theme	Description	*n*	Illustrative quotes
Medical experiences	Participants described often inconsistent care, late, missed or misdiagnosis, and a lack of professional knowledge of KS. Participants describe overall frustration with healthcare systems	12	‘I suffer more from issues around what is caused by my condition and what isn’t and then the endocrinology department I have are most unhelpful’
Infertility/family planning	Participants highlight a variety of emotional and psychological impacts from their infertility, including grief, identity concerns, and the relationship challenges faced. Further concerns were shaped around the complex interventions surrounding infertility	6	‘Finding out at 30 as a result of infertility was a massive psychological blow and it took me the best part of 10 years to come to terms with that’
			‘I felt it was easier to be on my own, which has led to an extremely depressing, lonely life, not being able to have children, sort of destroys the reason mankind exists’
Mental health	Participants described anxiety, depression and low mood, with existential hopelessness linked to delayed/fragmented care and uncertainty about the future	14	‘Having been diagnosed at younger age at time when information … had an impact on my sense of self-confidence and bodily esteem’
			‘KS/XXY gets me down quite a lot in life due to the nature of my body not working the same as other people’s my age’
Positive outlook/acceptance	Individuals express resilience and a positive mindset. Diagnosis brings self-awareness, connection with others, or personal growth	9	‘The diagnosis has made this easier to understand why I am the way I am. I’m very happy with my life and work and have a good strong support network’
Cognitive/learning difficulties	Focuses on difficulties with memory, concentration, academic performance, and information retention, described as often negatively affecting work or education	5	‘Found it harder when younger in education and making social friendship connections. Anxiety and learning difficulties’
Fatigue/low energy	Captures chronic exhaustion, reduced stamina, and limited ability to engage in work or physical activities. Often discussed in relation to testosterone levels or comorbid health issues	4	‘I have a limited amount of energy to carry out my work or leisure activity each day. And once it’s gone, it’s gone’
			‘Working is very difficult due to confusion and fatigue which I have been told is chronic and nothing nobody can do’

## Discussion

To our knowledge, this is the largest UK-based cohort to examine HR-QoL in adults with KS, demonstrating impairment across all domains of HR-QoL: physical health, psychological health, social relationships, and environment. Linear regression models identified perceived self-efficacy in clinical interactions as significantly associated with HR-QoL outcomes. Greater confidence in interactions with physicians, demonstrated by higher PEPPI-5 scores, was associated with increased HR-QoL scores in all domains.

Participants self-reported a variety of comorbidities, including high rates of neurodevelopmental disorders (33%), poor sleep quality (35%), gynaecomastia (35%), metabolic disorders/obesity (35%), and more than half of the cohort self-reported depression and/or anxiety (53%). These patterns were supported by the thematic analysis of open-ended questions, suggesting that the cumulative effects of comorbidities likely contribute to the observed HR-QoL deficits.

Although age and several sociodemographic characteristics were collected, this study was not designed to examine their independent associations with HR-QoL outcomes. Future research with larger cohorts or prospective design should investigate how these sociodemographic factors could influence or be associated with HR-QoL outcomes in adults with KS.

The included cohort had high levels of education: 36.7 and 21.7% obtained undergraduate and postgraduate degrees, respectively. While literature on educational attainment in people with Klinefelter syndrome is limited, previous studies examining QoL in KS have found lower levels of education achieved (4, 29). Consequently, the educational profile of this cohort may not be representative of the wider KS population. Despite the high levels of educational attainment in this cohort, HR-QoL deficits remained evident across the WHOQOL-BREF domains.

Perceived self-efficacy in patient–physician interactions was associated with WHOQOL-BREF scores across all domains: physical health, psychological health, social relations, and environment, both unadjusted and adjusted for comorbidity count, self-reported anxiety/depression, and BMI, suggesting that perceived self-efficacy in patient–physician interactions is associated with HR-QoL independently of physical health burden and mental health symptoms. These findings also suggest healthcare communications and perceived responsiveness in health services could be possible modifiable targets for improving HR-QoL outcomes in adults with KS.

The self-reported comorbidities and WHOQOL-BREF results point to clinically meaningful impairment. The large deficit in physical HR-QoL is consistent with prior literature describing a high burden of comorbidities in KS, including fatigue, disturbed sleep (sleep apnoea and/or poor sleep quality), and adverse cardiometabolic outcomes (30, 31). Similarly, lower psychological HR-QoL outcomes reported in this study echo previous research, which reports psychosocial difficulties, neurodevelopmental differences, and an increased mental health burden among individuals with KS (32, 33, 34, 35, 36). The WHOQOL-BREF survey results are also consistent with existing literature, indicating reduced HR-QoL in adults with KS (16, 37). In the large European multicentre DSD-LIFE cohort, Franik *et al.* reported significantly lower WHOQOL-BREF scores in the psychological, physical, and social domains when compared with healthy male reference data and controls, while the environment domain did not differ significantly (15). This aligns with an evidence synthesis showing that KS is commonly associated with reductions across physical, psychological, and social relationships and functioning (10).

Given that UK samples are underrepresented in the KS literature, the observed negative associations, particularly in the environment domain, may reflect country-specific health system factors and/or social attitudes towards KS instead of universal effects. Further investigation exploring whether these findings are geographically specific or universal would be advantageous in understanding the extent to which HR-QoL is influenced within this population.

The content analysis of open-ended responses contributes further support for the quantitative findings presented. The categories described, including low energy, adverse mental health, medical experiences, infertility, and family planning, align consistently with the deficits observed in the HR-QoL domains. These data reinforce the need for further in-depth qualitative exploration of the lived experiences of individuals with KS and the HR-QoL outcomes.

No significant differences in HR-QoL were reported between participants receiving TRT and those not receiving TRT across the WHOQOL-BREF domains, yet wide confidence intervals crossing zero may indicate imprecision. It is plausible that dosage variation, differing levels of adherence, and treatment duration could be influencing the observed group effects.

Two cross-sectional studies reported no significant differences in HR-QoL between adults with KS receiving TRT and those not receiving it, and no clear improvements in participants with KS treated with TRT compared with healthy controls (16, 38). These findings suggest that TRT alone may not be sufficient to improve HR-QoL, and that multifaceted interventions targeting the condition-specific nuances KS presents may be more relevant. However, previous studies were limited by small subgroup sizes, which restricted their power to detect smaller effects.

Self-perceived efficacy in clinical interaction scores highlights low self-perceived efficacy (14.52 ± 5.42). Participants felt least confident in ‘getting a clinician to take their chief health concerns seriously’, and most confident in ‘knowing what to ask your clinician’. This highlights a gap between participants’ ability to prepare for clinical encounters, knowing what to ask, and their confidence in those concerns being acknowledged. Previous studies using PEPPI-5 have reported higher participants’ confidence, with cross-sectional studies reporting median scores of 17–20 in other patient groups, suggesting that perceived self-efficacy in clinical interactions may be low in this cohort (39, 40, 41).

Notably, low self-perceived efficacy in clinical interactions had measurable consequences for HR-QoL (across all domains) in linear regression models. Thus, lower confidence in being heard and/or taken seriously in clinical encounters may contribute to broader impact on HR-QoL outcomes. This may reflect further barriers within healthcare/clinical interactions and service delivery, such as limited condition awareness or fragmented care, which could negatively contribute to well-being beyond clinical health status alone.

Adjusted analysis showed ∼4- to 7-point higher WHOQOL-BREF scores per 5-point higher PEPPI-5 scores; this was greatest for social and environment domains. This aligns with evidence examining self-efficacy in chronic disease populations, where patient co-creation and strategies to improve patients’ self-efficacy in collaboration with clinicians have been associated with improved clinical outcomes (19, 42). Qualitative accounts of limited KS awareness and fragmented care suggest possible modifiable health-service factors. Interventions targeting clinician education, structured consultation tools, care coordination, and greater peer support require further evaluation (18, 43).

The psychometric performance of the WHOQOL-BREF within this cohort was mixed. Internal consistency was acceptable to good for the physical, social, and environment domains, but poor for the psychological domain (*α* = 0.576). This lower internal consistency suggests reduced reliability of the psychological subscale in adults with KS, potentially reflecting limited content validity of the WHOQOL-BREF psychological items for KS-related concerns in a UK population.

It is well documented that individuals with KS exhibit considerable phenotypic heterogeneity, encompassing variations in reproductive, non-reproductive, physical, and psychological manifestations. Individuals may experience varying combinations and severities of symptoms, including executive function difficulties, social cognitive difficulties, stigma, infertility-related distress, neurodevelopmental delays, and chronic fatigue. Consequently, the findings reported in this study should be interpreted within the context of a broad clinical spectrum, as the experiences of individuals with KS may differ across the condition. Due to this, generic PROMs may not fully represent KS-related experiences. This further supports the development and validation of KS-specific outcome measures, alongside generic instruments.

Future research should incorporate longitudinal study designs to examine how clinical profiles, treatments, and healthcare experiences influence and shape HR-QoL over time. The use of objective outcomes, validated KS-specific PROMs, and the measurement of patient-physician engagement will help strengthen causal inference and inform targeted interventions.

### Strengths and limitations

The strengths of this study include providing UK-specific data on HR-QoL in adults with KS, addressing a gap in the current literature. Second, the use of validated instruments, including the PEPPI-5 and WHOQOL-BREF, allows for assessment of perceived self-efficacy in clinical interactions and HR-QoL. Third, comparison with pre-existing data and normative data allows for the extent of HR-QoL outcomes to be contextualised against population data. Finally, the inclusion of open-text responses within the survey helped provide additional qualitative insight into the individuals’ lived experiences, complementing the quantitative findings.

This study has several limitations. First, the cross-sectional study design limits causal inferences. Second, self-report of clinical variables (e.g. comorbidities, depression, anxiety) may introduce reporting bias. Third, TRT was recorded only as current use. Associations between TRT and HR-QoL should be viewed with caution as we collected limited information on regimen, dosage, adherence, treatment duration, or biochemical response. Fourth, adjusted analysis was restricted to complete cases (*n* = 55), which may reduce precision and could bias estimates. The final sample size (*n* = 60) may also limit statistical power and the generalisation of findings. While the pre-defined data-quality screening strengthened study validity, exclusion of 8 responses could have influenced the final cohort composition if those excluded participants differed from the retained sample. However, given the exploratory nature of the study, generalisation to the wider population was not a primary objective, and the findings should instead be interpreted as hypothesis-generating. Fifth, participants were recruited mainly through a UK patient support charity (KSA) via their membership list, social media, and patient meetings. As recruitment materials were distributed by KSA, rather than the research team, the number of individuals who received or viewed the study invitation was unknown, so formal participation and response rates could not be calculated. The recruitment process may have introduced recruitment bias, as individuals who actively engaged with support organisations may differ from the wider population of people with KS, including self-management strategies and engagement with KS-related services. In addition, voluntary participation may have introduced self-selection bias, as those who responded may have differed from non-responders in their motivation or experiences of KS. A larger study recruiting participants from endocrine centres, in addition to patient charities, is recommended. In addition, group comparisons involving the WHOQOL-BREF should be interpreted with caution, as the lower internal consistency observed in the psychological health domain items may not fully measure the more nuanced and specific symptoms and outcomes associated with the lived experiences of individuals with KS. Finally, the cohort was predominantly White and well educated. Thus, caution is warranted when attempting to generalise findings to historically minoritised and underrepresented groups.

## Conclusion

Adults with KS reported substantially reduced HR-QoL across physical, psychological, social, and environmental domains when compared with the UK normative data. Participants reported a variety of pre-existing health conditions, including high rates of depression, neurodevelopmental disorders, and adverse cardiometabolic health. Perceived self-efficacy in patient–physician interactions was consistently associated with better HR-QoL, suggesting that communication self-efficacy and health system responsiveness could be modifiable targets in improving HR-QoL outcomes.

These findings support the development of KS-informed care plans and pathways, clinical education resources, peer-support initiatives, and KS-specific outcome measures to capture the unique KS–psychological phenomenon experienced by individuals with KS and to work towards maximising HR-QoL outcomes in this population.

## Supplementary materials



## Declaration of interest

The authors declare that there is no conflict of interest that could be perceived as prejudicing the impartiality of the work reported.

## Funding

This research was supported by a research grant from the Endocrine Nurse Society (to BM).
