# Supplementary material for: A cross-sectional online survey of quality of life in adults with Klinefelter syndrome in the UK
Source: Endocr Connect. 2026 Oct 12;15(10):e260419. doi: 10.1530/EC-26-0419 (PMC13695396; doi:10.1530/EC-26-0419)
Supplement: Supplementary file 1 [file EC-26-0419_supplementary_materials.pdf]

Supplementary material

Table 1: STROBE Statement—Checklist of items that should be included in reports of ***cross-sectional studies***

	Item No	Recommendation
**Title and abstract**	1	(*a*) Indicate the study's design with a commonly used term in the title or the abstract. Lines: 1-2
		(*b*) Provide in the abstract an informative and balanced summary of what was done and what was found. L :27-47
**Introduction**		
Background/rationale	2	Explain the scientific background and rationale for the investigation being reported. L: 49-107
Objectives	3	State specific objectives, including any prespecified hypotheses. L: 105-107
**Methods**		
Study design	4	Present key elements of study design early in the paper. L: 109-114
Setting	5	Describe the setting, locations, and relevant dates, including periods of recruitment, exposure, follow-up, and data collection. L: 120-122
Participants	6	(*a*) Give the eligibility criteria, and the sources and methods of selection of participants. L: 115-119
Variables	7	Clearly define all outcomes, exposures, predictors, potential confounders, and effect modifiers. Give diagnostic criteria, if applicable. L:124-143
Data sources/ measurement	8*	For each variable of interest, give sources of data and details of methods of assessment (measurement). Describe comparability of assessment methods if there is more than one group. L: 124-143
Bias	9	Describe any efforts to address potential sources of bias. L:145-153
Study size	10	Explain how the study size was arrived at. L145-153
Quantitative variables	11	Explain how quantitative variables were handled in the analyses. If applicable, describe which groupings were chosen and why. L139-143 & 159-167
Statistical methods	12	(*a*) Describe all statistical methods, including those used to control for confounding. L:150-172 (all analysis described)
		(*b*) Describe any methods used to examine subgroups and interactions. L:150-172 (all analysis described)
		(*c*) Explain how missing data were addressed. Missing data indicated by n values in parentheses throughout tables.
		(*d*) If applicable, describe analytical methods taking account of sampling strategy. N/A
		(*e*) Describe any sensitivity analyses. L272-276
**Results**		
Participants	13*	(a) Report numbers of individuals at each stage of study—eg numbers potentially eligible, examined for eligibility, confirmed eligible, included in the study, completing follow-up, and analysed. L:148-150, (each table clearly defines participant (n), as well as demographics table included).
		(b) Give reasons for non-participation at each stage. L148-150

		(c) Consider use of a flow diagram N/A
Descriptive data	14*	(a) Give characteristics of study participants (eg demographic, clinical, social) and information on exposures and potential confounder. Table 1 provides study demographics. Table 2: TRT and fertility. and Figure 2: comorbidity profile including key confounders (depression/anxiety 53%).
		(b) Indicate number of participants with missing data for each variable of interest '(n) values provided'
Outcome data	15*	Report numbers of outcome events or summary measures. Cronbach's α per WHOQOL domain (physical α=0.858, psychological α=0.576, social α=0.746, environment α=0.848); WHOQOL-BREF domain means (SD) for N=60 reported. Group means vs. normative data in figure 2; TRT vs. no-TRT in figure 3.
Main results	16	(*a*) Give unadjusted estimates and, if applicable, confounder-adjusted estimates and their precision (eg, 95% confidence interval). Make clear which confounders were adjusted for and why they were included. Table 5 & L. 161-167
		(*b*) Report category boundaries when continuous variables were categorized. N/A
		(*c*) If relevant, consider translating estimates of relative risk into absolute risk for a meaningful time period. N/A
Other analyses	17	Report other analyses done—eg analyses of subgroups and interactions, and sensitivity analyses 'All analysis described and reported in main text. L: 210-231, 246-252, & 283-304
**Discussion**		
Key results	18	Summarise key results with reference to study objectives L: 309-318
Limitations	19	Discuss limitations of the study, taking into account sources of potential bias or imprecision. Discuss both direction and magnitude of any potential bias L: 430-438
Interpretation	20	Give a cautious overall interpretation of results considering objectives, limitations, multiplicity of analyses, results from similar studies, and other relevant evidence L: 474-485
Generalisability	21	Discuss the generalisability (external validity) of the study results.
**Other information**		
Funding	22	Give the source of funding and the role of the funders for the present study and, if applicable, for the original study on which the present article is based. L:18-19

*Give information separately for exposed and unexposed groups.

Note: An Explanation and Elaboration article discusses each checklist item and gives methodological background and published examples of transparent reporting. The STROBE checklist is best used in conjunction with this article (freely available on the Web sites of PLoS Medicine at http://www.plosmedicine.org/, Annals of Internal Medicine at http://www.annals.org/, and Epidemiology at http://www.epidem.com/). Information on the STROBE Initiative is available at www.strobe-statement.or

Table 2. WHOQOL-BREF domain scores in adults with Klinefelter syndrome
2A. Participants with KS vs UK pre-existing normative population data.

QoL domain	KS Participants (N= 60)	UK Controls (N= 141)	*P* value	Effect size (*d*)
Physical mean (SD)	*53.50 (5.92)*	*75.41 (18.72)*	*<0.0001*	*-1.37*
Psychological mean (SD)	*51.97 (11.50)*	*70.21 (15.84)*	*<0.0001*	*-1.24*
Social mean (SD)	*53.78 (14.56)*	*71.37 (19.82)*	*<0.0001*	*-0.96*
Environmental mean (SD)	*60.47 (17.95)*	*72.26 (14.78)*	*<0.0001*	*-0.75*

2B. Participants KS with TRT vs KS without TRT

QoL domain	KS with TRT (N= 44)	KS without TRT (N= 16)	*P* value	Effect size (*d*)
Physical mean (SD)	*54.98 (11.27)*	*49.44 (11.52)*	*0.10*	*0.49*
Psychological mean (SD)	*53.46 (13.52)*	*47.88 (16.91)*	*0.20*	*0.36*
Social mean (SD)	*53.27 (26.80)*	*55.17 (21.52)*	*0.80*	*-0.09*
Environmental mean (SD)	*62.15 (17.65)*	*55.85 (18.55)*	*0.23*	*0.35*

***Footnote**: The internal consistency (Cronbach's a) was measured for each domain of the WHOQOL within this studies population (n=60), results were physical a = 0.858, psychological a= 0.576, social a = 0.746, environment a = 0.848.*

Sample Qualtrics survey

This survey has been exported from Qualtrics; content remains the same, but formatting has been adapted for clarity.

Survey information

Welcome to this research study!

Study title: Quality of Life in Adults with Klinefelter's Syndrome / XXY: Questionnaire survey

Principal Investigator: Mr Brien Mehmet

Before deciding to participate, please read the Patient Information Sheet linked below to understand the purpose and requirements of this research. You may discuss it with others if you wish.

For questions or clarification, contact Mr. Brien Mehmet at brien.mehmet@city.ac.uk. You can also download a PDF of this information for future reference. The survey takes around 20 minutes. For each completed questionnaire, £2 will be donated to the Klinefelter's Syndrome Association (KSA).

Please complete it in one session, if possible. However, you may save and return to it if needed. By submitting the survey, you consent to your responses being used in this research. Due to its anonymous nature, responses cannot be withdrawn once submitted. Survey completion indicates consent.

KKSQOL Participant Information Sheet

KSQOL Participation Information Sheet (accessible)

Country status

Do you currently live in the United Kingdom?

☐ Yes	☐ No

If no: Thank you for taking part! Unfortunately, this survey is currently for UK participants only at this moment. We plan to launch an international survey soon. Please contact brien.mehmet@city.ac.uk if you have any further questions or would like updates on the international survey.

Eligibility criteria

Eligibility checklist: Please read the following statements and confirm if they apply to you by ticking each box. You must meet all the eligibility criteria below to participate in this research study.

☐ I confirm that I am 18 years of age or older.
☐ I confirm that I have read the participant information sheet, I understand what the study involves and am happy to proceed with the survey.
☐ I confirm that I have a satisfactory level of English to understand and complete the survey or have someone with me who does (all answers must remain those of the participant).
☐ I confirm that I will not provide any personal details (name or contact number/email) in this survey as it is anonymous with no possibility to identify individual responses.
☐ I confirm I have been diagnosed with Klinefelter Syndrome / XXY.

Section 1 - Information about you, your diagnosis and your treatment

Please tell us your age range from the options below.

☐ 18-24	☐ 25-34	☐ 35-44
☐ 45-54	☐ 55-64	☐ 65 and over

How old were you when you were diagnosed with Klinefelter's Syndrome / XXY?

____

Please tell us how you identify.

☐ Male	☐ Female
☐ Non-binary / third gender	☐ Prefer not to say
☐ Other (please specify)	

What best describes your ethnic origin?

☐ White	☐ Black/African/Caribbean
☐ Asian (Indian, Pakistani, Bangladeshi, Chinese, any other Asian background)	☐ Mixed two or more ethnic groups
☐ Other (Arab or any others)	☐ Prefer not to say

What is the highest level of education you have received?

☐ Did not obtain GCSE or O levels	☐ Secondary school / High School (GCSE/O levels)
☐ Undergraduate degree	☐ Postgraduate degree

What is your marital status?

☐ Single	☐ Married (including civil partnership)
☐ In a relationship	☐ Separated
☐ Divorced	☐ Widowed
☐ Prefer not to say	

What is your height in (please specify) Centimeters or Feet and Inches?

____

What is your current weight in (please specify) Kilograms or Stone and lbs?

____

Please state if you have any other health issues or conditions by ticking all that apply from the list below.

☐ Negative bone health (frequent fractures or osteoporosis)	☐ Male breasts / gynaecomastia
☐ Cancers (all types)	☐ Epilepsy
☐ Depression / Anxiety	☐ Diabetes
☐ Neurodivergent diagnoses (please state below), including autism spectrum disorder, dyslexia, dyspraxia, Tourette's syndrome	☐ Obesity
☐ Blood clots / Thrombosis	☐ Pulmonary embolism

☐ Anaemia	☐ Cardiac disease / heart attack
☐ Asthma / COPD / respiratory problems	☐ Sleep apnoea
☐ Poor sleep quality	☐ Other (please specify)

Testosterone Replacement Therapy

Are you currently taking testosterone?

☐ Yes	☐ No

Have you ever been offered testosterone?

☐ Yes	☐ No

If yes, which type of testosterone are you taking?

☐ Applied to skin 50-100 mg of 1% Testogel once daily
☐ Applied to skin 40-70 mg of 2% Tostran once daily
☐ Applied to skin 23-46 mg of Testavan once daily
☐ Applied to skin 20.25-81 mg of 16.2 mg/g (Testogel pump) transdermal gel once daily
☐ Long-acting injection 1000 mg in 4 ml (Nebido) oily preparation every 10-14 weeks
☐ Short acting injection Testosterone esters 250 mg/ml (Sustanon) IM every 3-4 weeks
☐ Testosterone enanthate or cypionate 150-200 mg IM every 2 weeks or 50-100 mg IM or SC once a week
☐ Testosterone pellets 100 or 200 mg
☐ Oral T undecanoate capsules (40 mg)
☐ Other (please specify)

Employment

Which following best described your work life?

☐ I am currently or was previously employed
☐ I am in long term unemployment
☐ I am a full-time student
☐ Retired
☐ Prefer not to say

Fertility

This section will involve answering questions regarding your fertility; some questions may be sensitive. Please mark as "prefer not to say" if you do not want to complete these questions.

Have you attempted fertility treatment?

☐ Yes	☐ No	☐ Prefer not to say

Do you have children?

☐ Yes	☐ No	☐ Prefer not to say

How did you become a parent? Please select most appropriate answer.

☐ Conceived naturally	☐ Adopted
☐ Assisted fertility	☐ Sperm donation

☐ Prefer not to say	☐ Other (please specify)

Perceived Efficacy in Patient-Physician Interactions questionnaire (short form)

The next section is regarding your own experiences when attending or speaking to clinicians. This can involve going to your GP or specialist clinics. Please tick the right box under the scale: not at all confident to extremely confident.

How confident are you in your ability to?

**Question**	**Not at all confident**	**Somewhat confident**	**Unsure**	**Confident**	**Very confident**
Know what questions to ask a clinician	☐	☐	☐	☐	☐
Get a clinician to answer all of your questions	☐	☐	☐	☐	☐
Make the most of your visit with the clinician	☐	☐	☐	☐	☐
Get a clinician to take your chief health concerns seriously	☐	☐	☐	☐	☐
Get a clinician to do something about your chief health concern	☐	☐	☐	☐	☐

WHOQOL–BREF

UK VERSION

Department of Mental Health

World Health Organisation

Geneva

Instructions

This questionnaire asks how you feel about your quality of life, health and other areas of your life. **Please answer all the questions**. If you are unsure about which response to give to a question, **please choose the ONE** that appears most appropriate. This can often be your first response.

Please keep in mind your standards, hopes, pleasures and concerns. We ask that you think about your life **in the last two weeks**. For example, thinking about the last two weeks, a question might ask:

		Not at all	Not much	Moderately	A great deal	Completely
	Do you get the kind of support from others that you need?	1	2	3	4	5

You should **circle** the number that best fits how much support you got from others **over the last two weeks**. So you would circle the number 4 if you got a great deal of support from others as follows:

		Not at all	Not much	Moderately	A great deal	Completely
	Do you get the kind of support from others that you need?	1	2	3	4	5

You would circle the number 1 if you did not get any of the support that you needed from others in the last two weeks. Please read each question, assess you feelings, and **circle** the number on the scale for each question that gives the best answer for you.

	**Very poor (1)**	**Poor (2)**	**Neither poor nor good (3)**	**Good (4)**	**Very good (5)**
How would you rate your quality of life?	☐	☐	☐	☐	☐

	**Very dissatisfied (1)**	**Dissatisfied (2)**	**Neither satisfied nor dissatisfied (3)**	**Satisfied (4)**	**Very satisfied (5)**
How satisfied are you with your health?	☐	☐	☐	☐	☐

The following questions ask about how much you have experienced certain things in the last two weeks.

	**Not at all (1)**	**A little (2)**	**A moderate amount (3)**	**Very much (4)**	**An extreme amount (5)**
To what extent do you feel that physical pain prevents you from doing what you need to do?	☐	☐	☐	☐	☐
How much do you need medical treatment to function in your daily life?	☐	☐	☐	☐	☐
How much do you enjoy life?	☐	☐	☐	☐	☐
To what extent do you feel your life to be meaningful?	☐	☐	☐	☐	☐

The following questions ask about how completely you experience or were able to do certain things in the last two weeks.

	Not at all (1)	A little (2)	A moderate amount (3)	Very much (4)	Extremely (5)
How well are you able to concentrate?	☐	☐	☐	☐	☐
How safe do you feel in your daily life?	☐	☐	☐	☐	☐
How healthy is your physical environment?	☐	☐	☐	☐	☐
Do you have enough energy for everyday life?	☐	☐	☐	☐	☐
Are you able to accept your bodily appearance?	☐	☐	☐	☐	☐
Have you enough money to meet your needs?	☐	☐	☐	☐	☐
How available to you is the information that you need in your day-to-day life?	☐	☐	☐	☐	☐
To what extent do you have the opportunity for leisure activities?	☐	☐	☐	☐	☐

	Very poor (1)	Poor (2)	Neither poor nor good (3)	Good (4)	Very good (5)
How well are you able to get around?	☐	☐	☐	☐	☐

The following questions ask you how good or satisfied you have felt about various aspects of your life over the last two weeks.

	Very dissatisfied (1)	Dissatisfied (2)	Neither dissatisfied nor satisfied (3)	Satisfied (4)	Very satisfied (5)
How satisfied are you with your sleep?	☐	☐	☐	☐	☐
How satisfied are you with your ability to perform your daily living activities?	☐	☐	☐	☐	☐
How satisfied are you with your capacity for work?	☐	☐	☐	☐	☐
How satisfied are you with yourself?	☐	☐	☐	☐	☐
How satisfied are you with your personal relationships?	☐	☐	☐	☐	☐
How satisfied are you with your sex life?	☐	☐	☐	☐	☐
How satisfied are you with the support you get from your friends?	☐	☐	☐	☐	☐
How satisfied are you with the conditions of your living place?	☐	☐	☐	☐	☐

How satisfied are you with your access to health services?	☐	☐	☐	☐	☐
How satisfied are you with your transport?	☐	☐	☐	☐	☐

The following question refers to how often you have felt or experienced certain things in the last two weeks.

	**Never (1)**	**Seldom (2)**	**Quite often (3)**	**Very often (4)**	**Always (5)**
How often do you have negative feelings such as blue mood, despair, anxiety, depression?	☐	☐	☐	☐	☐

Did someone help you to fill out this form?

☐ Yes	☐ No

Additional comments

Feel free to share how Klinefelter's syndrome / XXY has impacted your quality of life, well-being, or any other aspects, along with any additional comments or experiences not covered in this questionnaire.
